# Supplementary material for: Glycan shield of the ebolavirus envelope glycoprotein GP
Source: Commun Biol. 2022 Aug 4;5:785. doi: 10.1038/s42003-022-03767-1 (PMC9352669; doi:10.1038/s42003-022-03767-1)
Supplement: Supplementary file 1 — Supplementary Information [file 42003_2022_3767_MOESM1_ESM.pdf]

Supplementary Information to:

Glycan shield of the ebolavirus envelope glycoprotein GP

Weiwei Peng [1], Vamseedhar Rayaprolu [2,4], Amar D. Parvate [2,5], Matti F. Pronker [1], Sean Hui [2,6], Diptiben Parekh [2], Kelly Shaffer [2,7], Xiaoying Yu [2], Erica O. Saphire [2,3], Joost Snijder [1]*

[1] Biomolecular Mass Spectrometry and Proteomics, Bijvoet Center for Biomolecular Research and Utrecht Institute of Pharmaceutical Sciences, Utrecht University, Padualaan 8, 3584 CH Utrecht, The Netherlands

[2] Center for Infectious Disease and Vaccine Discovery, La Jolla Institute for Immunology, La Jolla, CA 92037, USA.

[3] Department of Medicine, University of California, San Diego, La Jolla, CA 92039, USA

[4] Current affiliation - Pacific Northwest Center for CryoEM, Portland, OR 97225, USA

[5] Current affiliation - Environmental Molecular Sciences Laboratory, Pacific Northwest National Laboratory, Richland, WA 99354, USA

[6] Current affiliation – Molecular Microbiology and Microbial Pathogenesis Program, Washington University School of Medicine, Saint Louis, MO 63108, USA

[7] Current affiliation – Division of Biology and Biological Engineering, California Institute of Technology, Pasadena, CA 91125, USA

*corresponding author: j.snijder@uu.nl

Supplementary Figures S1-S5

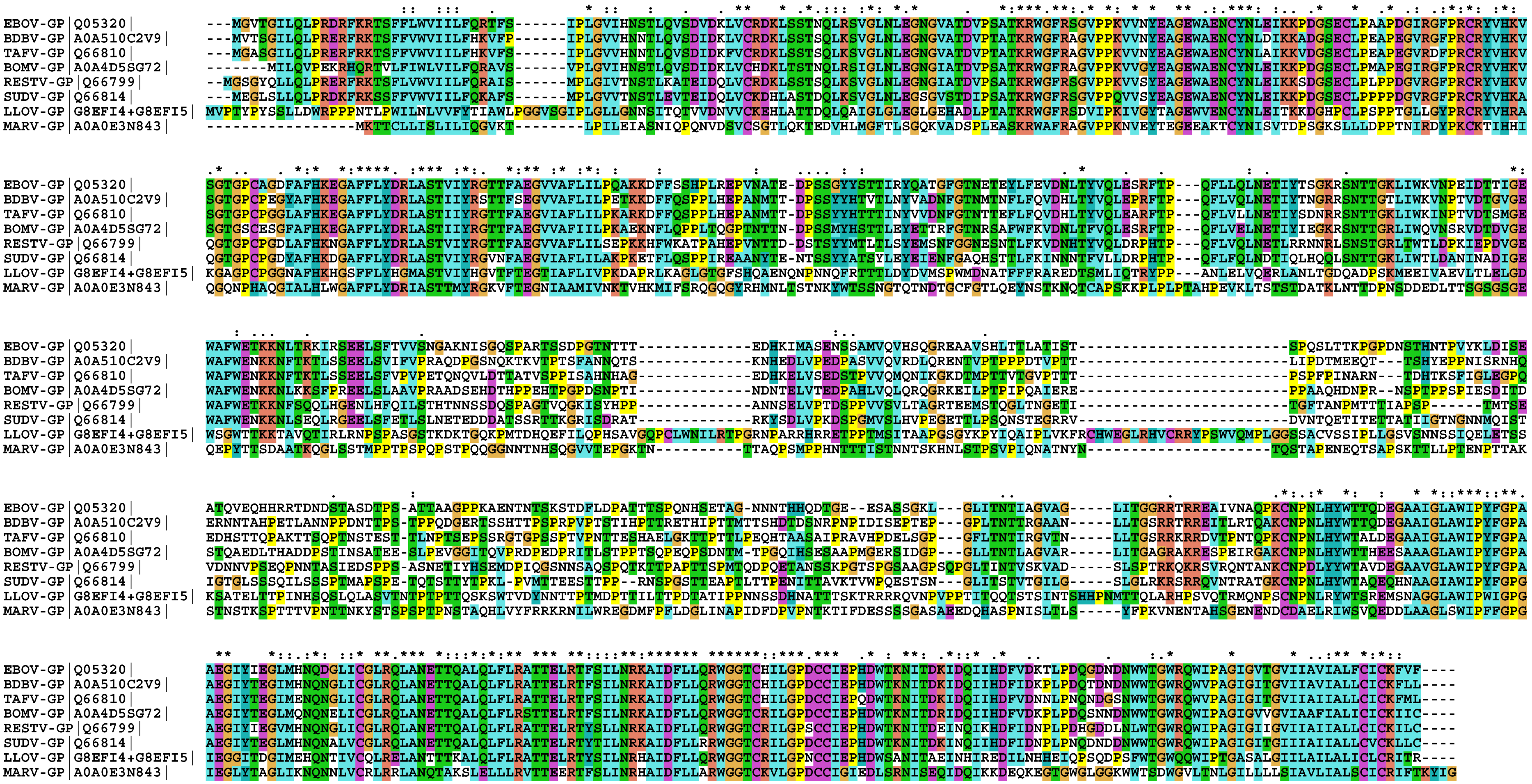

Supplementary Figure S1. Multiple sequence alignment of filovirus GP used for the analyses presented in main text (Figure 1). Corresponding UniProt IDs are provided in the sequence names.

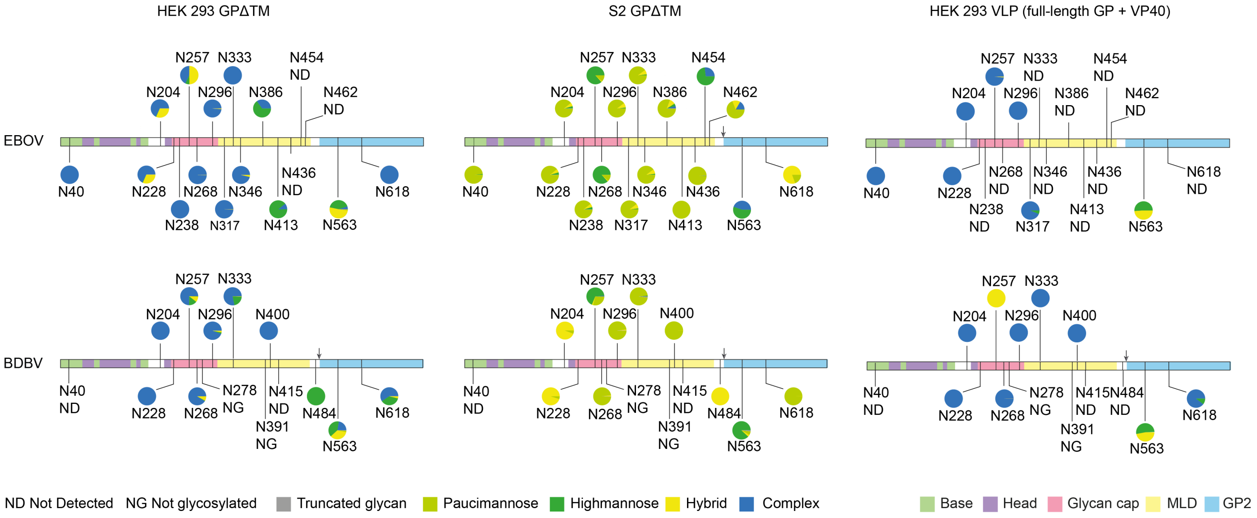

Supplementary Figure S2. Overview of site-specific N-linked glycan processing in ebola virus GPΔTM from HEK293 and S2 cells, versus full-length GP from HEK293 derived VLPs as determined by LC-MS/MS. The glycans were classified by HexNAc content as truncated, paucimannose, high-mannose, hybrid or complex. Shown is the average of a duplicate experiment.

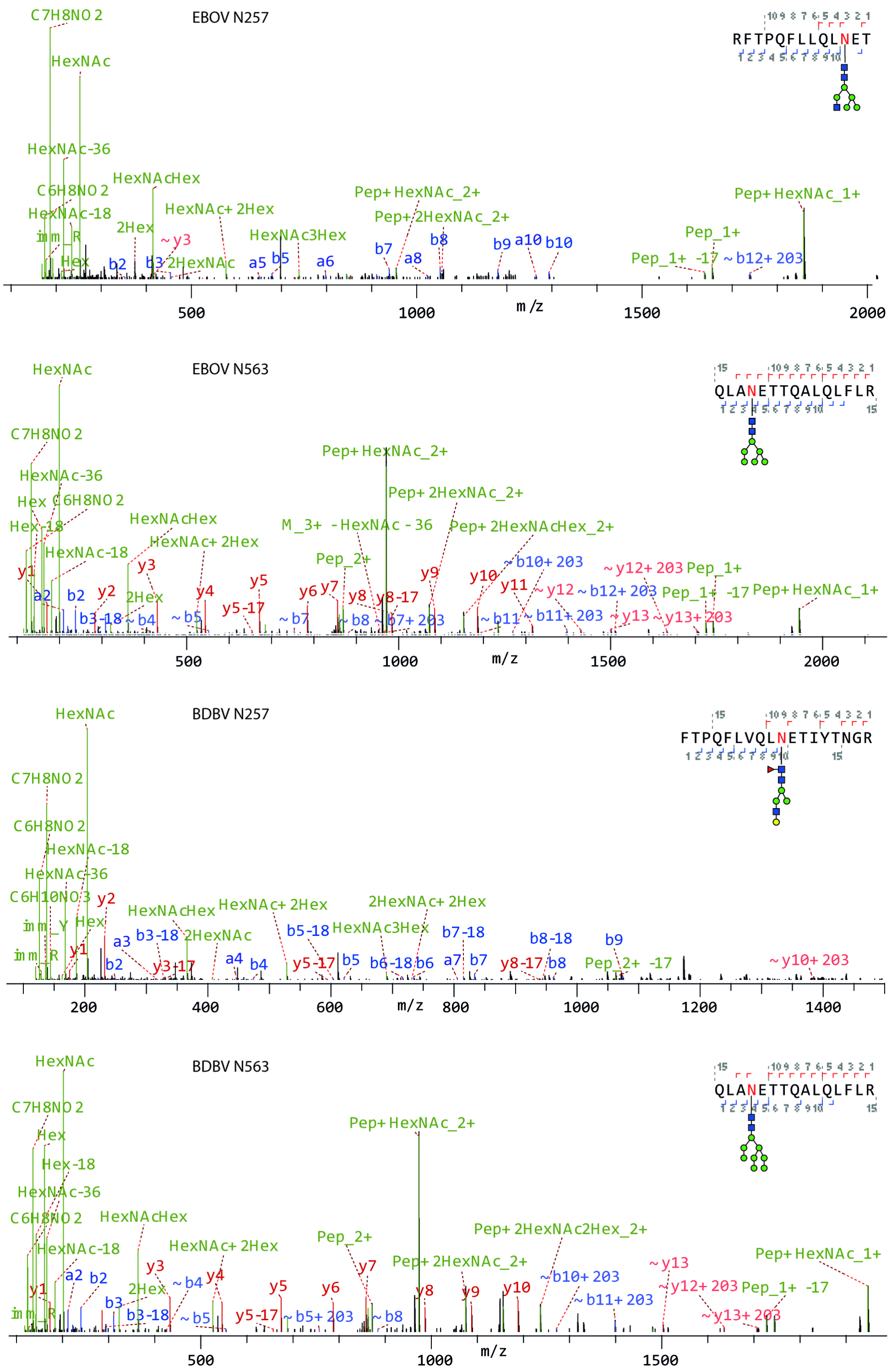

Supplementary Figure S3. MS/MS spectra of glycopeptides containing sites N257 or N563 of full-length EBOV and BDBV GP from HEK293 derived VLPs with unprocessed high-mannose and hybrid glycans.

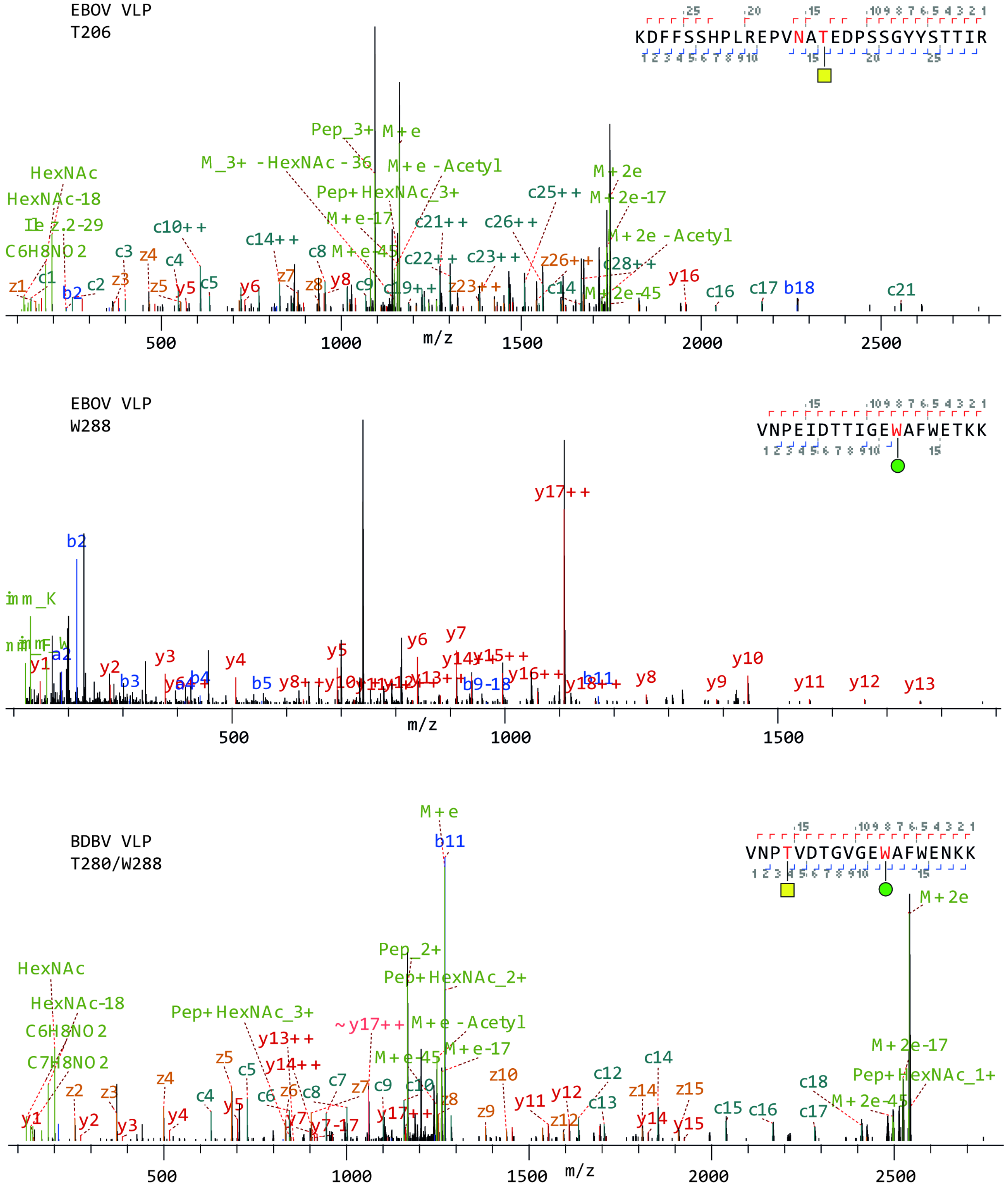

Supplementary Figure S4. MS/MS spectra of glycopeptides containing sites O-linked glycosylation outside the MLD and C-linked mannosylation at W288 in full-length EBOV and BDBV GP from HEK293 derived VLPs with unprocessed high-mannose and hybrid glycans.

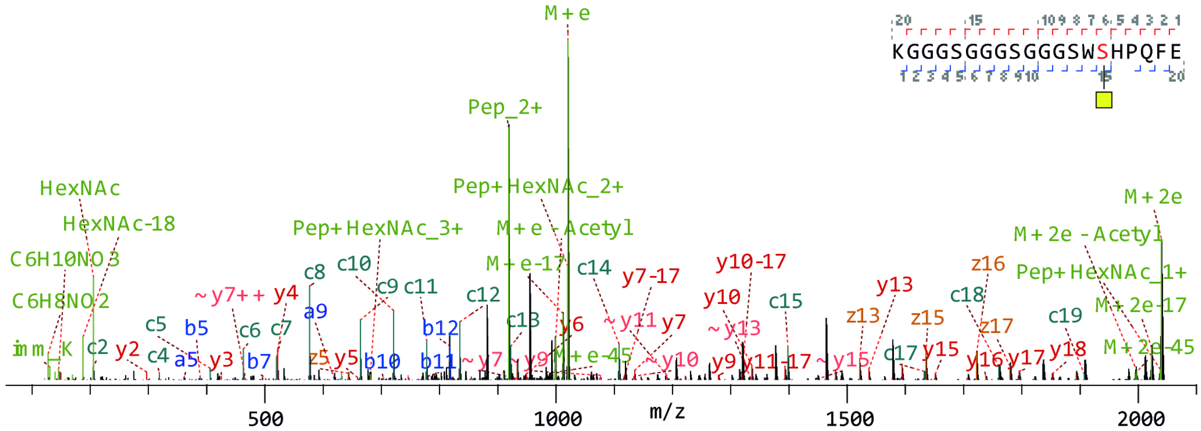

Supplementary Figure S5. MS/MS spectrum of glycopeptide from HEK293-derived BDBV GPΔTM with O-linked glycosylation in the Strep-tag of the recombinant soluble ectodomain.
