# Supplementary material for: Glycan shield of the ebolavirus envelope glycoprotein GP
Source: Commun Biol. 2022 Aug 4;5:785. doi: 10.1038/s42003-022-03767-1 (PMC9352669; doi:10.1038/s42003-022-03767-1)
Supplement: Supplementary file 2 — Description of Additional Supplementary Files [file 42003_2022_3767_MOESM2_ESM.pdf]

Description of Additional Supplementary Files

File name: Supplementary Data S1
Description: Lists of site-specific N-linked glycan compositions identified with Byonic from LC-MS/MS of EBOV and BDBV GP (for GPΔTM and VLP), alongside fractional peak areas for both MS runs. Each sample type (HEK293/S2, EBOV/BDBV, GPΔTM/full-length) is provided in a separate file, with a complete list on the first tab and the individual sites provided separately on subsequent tabs. Abbreviations: HexNAc (N-acetylated Glucosamine), Hex (Hexose), Fuc (Fucose), NeuAc (Nacetyl Neuraminic acid).
